# Attention, sentiments and emotions towards emerging climate technologies on Twitter

**DOI:** 10.1016/j.gloenvcha.2023.102765

**Published:** 2023-12

**Authors:** Finn Müller-Hansen, Tim Repke, Chad M. Baum, Elina Brutschin, Max W. Callaghan, Ramit Debnath, William F. Lamb, Sean Low, Sarah Lück, Cameron Roberts, Benjamin K. Sovacool, Jan C. Minx

**Affiliations:** a Mercator Research Institute on Global Commons and Climate Change (MCC), Germany; b Department of Business Technology and Development, Aarhus University, Denmark; c International Institute for Applied Systems Analysis (IIASA), Austria; d Cambridge Collective Intelligence & Design Group, Cambridge Zero and Computer Laboratory, University of Cambridge, United Kingdom; e Division of Humanities and Social Science, California Institute of Technology (Caltech), USA; f Centre for Sustainability and the Global Environment (SAGE), University of Wisconsin Madison, USA; g Priestley International Centre for Climate, University of Leeds, United Kingdom; h Science Policy Research Unit (SPRU), University of Sussex Business School, United Kingdom; i Department of Earth and Environment, Boston University, United States

**Keywords:** Public perception, Geoengineering, Solar radiation management, Greenhouse gas removal, Social media, Sentiment analysis

## Abstract

Public perception of emerging climate technologies, such as greenhouse gas removal (GGR) and solar radiation management (SRM), will strongly influence their future development and deployment. Studying perceptions of these technologies with traditional survey methods is challenging, because they are largely unknown to the public. Social media data provides a complementary line of evidence by allowing for retrospective analysis of how individuals share their unsolicited opinions. Our large-scale, comparative study of 1.5 million tweets covers 16 GGR and SRM technologies and uses state-of-the-art deep learning models to show how attention, and expressions of sentiment and emotion developed between 2006 and 2021. We find that in recent years, attention has shifted from general geoengineering themes to specific GGR methods. On the other hand, there is little attention to specific SRM technologies and they often coincide with conspiracy narratives. Sentiments and emotions in GGR tweets tend to be more positive, particularly for methods perceived to be natural, but are more negative when framed in the geoengineering context.

## Highlights

• We analyze and compare tweets on geoengineering and 16 related climate technologies.
• Attention has shifted from general geoengineering to specific carbon removal methods.
• Sentiments are more positive for carbon removal than solar radiation management.
• Methods perceived closer to nature have the highest shares of positive sentiments.
• Our social media analysis is consistent with survey results and qualitative research.

## 1 Introduction

Geoengineering can be defined as the large-scale, intentional intervention into the climate system to counteract anthropogenic climate change (Keith, 2000, The Royal Society, 2009). In this context, two sets of technologies and methods have been discussed: solar radiation management (SRM) and greenhouse gas removal (GGR). While GGR technologies address the cause of climate change by removing greenhouse gases from the atmosphere (Minx et al., 2018), SRM methods aim to reduce global warming and climate impacts by reflecting some of the sunlight’s energy away from the earth (National Academies of Sciences, 2021). While some GGR methods such as afforestation are widely practiced, most GGR and SRM technologies are still at the conceptual R&D stage or in early demonstration and deployment stages.

Both sets of methods have been discussed, at times controversially, within both science and policy circles (Schellnhuber, 2011, Anderson and Peters, 2016, Keith, 2021). Assessments highlight fundamental differences in the risks associated with planetary scale deployments: The sixth assessment report by IPCC Working Group III emphasizes that limiting global warming to well below 2°C requires large-scale GGR deployment in addition to ambitious mitigation of greenhouse gas emissions and that net-zero commitments by countries imply some level of GGR (IPCC, 2022). As climate impacts will surge in the future, SRM will be increasingly discussed as a solution, with huge challenges for international governance (Aldy et al., 2021, Abatayo et al., 2020, Emmerling and Tavoni, 2018).

Even though discussions about GGR and SRM have so far been driven by scientists and policymakers, it is crucial to engage with the public (Buck, 2016, Bellamy, 2018, Carr et al., 2013). Understanding public attitudes and concerns about emerging SRM and GGR technologies as well as engaging citizens in deliberations about their deployment at scale can help prevent or mitigate potential societal conflict (Colvin et al., 2020, Forster et al., 2020). How technology adopters and the wider public perceive those technologies will influence the prospects for researching, developing and scaling them up (Nemet et al., 2018, Cox et al., 2020). For example, past experience has shown that even small-scale scientific projects have been canceled due to strong public opposition, possibly caused by inadequate communication by and misinformation about the projects. At the same time, from an ethical and justice-relevant perspective, understanding public perceptions on integrating a broader range of individuals and perspectives, if done well, can support responsible innovation efforts of these new and emerging technologies (Bellamy, 2018, Low and Buck, 2020).

Surveys have found that the general population knows very little about GGR technologies (Cox et al., 2020, Pianta et al., 2021, Pidgeon and Spence, 2017, Sweet et al., 2021, Whitmarsh et al., 2019) and even less about SRM (Mercer et al., 2011). Perceptions of GGR and SRM technologies range widely, from support to entrenched opposition as they are perceived to threaten near-term climate action, have harmful side effects, or can even be seen as evidence of global conspiracies. A richer understanding of how different technologies are perceived emotionally is important, because emotions and worldviews play a key role in the perception of technologies and for climate policy support (Martiskainen and Sovacool, 2021, Duggan et al., 2021, Wang et al., 2018, Visschers et al., 2017). Furthermore, worldviews may lead to the rejection of scientific insights and recommendations (Lewandowsky et al., 2013, Smith and Leiserowitz, 2014), making it difficult to cultivate an informed public deliberation about the application of SRM and GGR technologies and methods as well as the kinds of risk, benefits, challenges, and opportunities associated with them (Sovacool, 2021).

Revealing public perceptions of new and emerging technologies using methods such as surveys, experiments or deliberative approaches can be challenging. The lack of familiarity of respondents with these technologies can result in framing effects and elicited perceptions being malleable and readily influenced by the questionnaire (Whitmarsh et al., 2019, Raimi, 2021, Lenzi, 2019). Moreover, comparative studies are usually restricted to three or four GGR or SRM methods and often to one or few countries and points in time (Lewandowsky et al., 2013, Spence et al., 2021, Carlisle et al., 2020, Bellamy et al., 2019, Jobin and Siegrist, 2020, Cox et al., 2020). Surveys and qualitative studies are able to provide information on representative samples of a population or details about psychological mechanisms. However, they are often limited to only studying a small number of technologies, thereby making comparative assessments across technologies difficult.

In contrast, social media data can help to understand the communicative and temporal aspects of a potentially large number of new and emerging technologies (Cortis and Davis, 2021). Public conversations on emerging technologies on social media provide an opportunity to investigate how people engage with these topics without being asked. Contrary to elicited opinion polls, social media analyses can capture various aspects that users associate with those technologies without being influenced by the way certain questions are posed. In the context of geoengineering, this is a key advantage because studies have highlighted the importance of framing and with that introducing a bias especially for little-known technologies (Pidgeon and Spence, 2017, Whitmarsh et al., 2019, Bäckstrand et al., 2011, Wolske et al., 2019). This data thus allows identifying the status and evolution of general trends in attitudes and emotions towards them. At the same time, user interaction is influenced by algorithmic ranking mechanisms that amplify users’ exposure to specific content (Huszár et al., 2022, Sen et al., 2021). It must also be recognised that users are not representative of the general population (Mellon and Prosser, 2017, Barberá and Rivero, 2015, Wojcik et al., 2019). User activity on the platform is strongly dominated by few very active users, who are often professional communicators from politics, business, journalism and science. These may include stakeholder groups relevant for developing and deploying emerging technologies in the future.

Social media analyses can therefore complement the survey literature and inform policy makers and technology proponents regarding how to engage in debates and how to resolve potential conflicts early on. Social media and in particular Twitter^1^ data has been used in the literature to study attention, sentiments and emotions on climate change in general (Cody et al., 2015, Kirilenko et al., 2015, Moore et al., 2019, Falkenberg et al., 2022, Mouronte-López and Subirán, 2023), in times of the Covid-19 pandemic (Smirnov and Hsieh, 2022, Sisco et al., 2023), as well as related to energy technologies (Loureiro and Alló, 2020). So far, there are only two social media analyses on general and solar geoengineering (Tingley and Wagner, 2017, Debnath et al., 2023), which find a high prevalence of misinformation and tweets spreading conspiracy theories. However, none of them looks into specific geoengineering technologies.

In this paper, we present an analysis of 1.5 million tweets related to general geoengineering, GGR and SRM topics as well as a total of 16 SRM and GGR technologies. We compile a dataset based on 78 individual keyword searches that cover the entire history of Twitter, starting in 2006. By comparing tweet counts, we quantify how attention to different SRM and GGR technologies has evolved over time. We apply state-of-the-art deep learning models based on BERTweet (Nguyen et al., 2020) and other RoBERTa models (Liu et al., 2019) to identify topical clusters and measure sentiments and emotions. We validate classifiers against each other and use manual annotations to ensure the robustness of our results. This allows us to compare sentiments and emotions towards different technologies. Finally, we investigate how ubiquitous conspiracy theories are for different technologies. In summary, we map out trends in social media activity on geoengineering and show parallels as well as differences to findings from survey-based methods.

## 2 Material and methods

### 2.1 Twitter dataset

The data gathering and processing for this paper was conducted closely following ethical recommendations for using semi-public social media data for empirical research (Williams et al., 2017) and adhering to Twitter’s Terms of Service. We develop a comprehensive keyword search and download tweets through the Twitter API v2, giving us access to the entire database of tweets back to 2006. In total, we search Twitter using 78 distinct sub-queries (see Supporting Information). Each sub-query contains a keyword or combination of keywords. We download tweets separately for each sub-query, excluding non-English tweets, retweets, and very recent tweets after December 31, 2021.

We carefully check a random sample from each sub-query to make sure that it largely yields tweets that are relevant to the study, i.e. that talk about geoengineering, modifying the climate or removing carbon from the atmosphere. In an iterative process, we refine or exclude sub-queries to get higher shares of relevant tweets (at least 80%). If the proportion of irrelevant tweets is high (e.g. because an acronym is used differently or serves as a stock market ticker), we restrict the query by excluding tweets with certain unrelated keywords or requiring that more general keywords such as “climate”, “emission” or “CO2” are mentioned as well. If sub-queries did hardly yield any relevant results, we removed them. This refinement of sub-queries allows us to reduce noise in our dataset.

Sub-queries are organized into categories at two levels. At the top level, we group tweets into “Geoengineering” if they contain general geoengineering keywords, into “SRM” if they contain keywords related to solar radiation management in general or specific technologies for implementing SRM, “GGR” if they discuss greenhouse gas removal and related technologies and “CCS” if they talk about carbon capture and storage. CCS refers to technologies to capture carbon from exhaust gases of fossil combustion, which is why it is not a GGR technique. However, it is closely related to many GGR techniques like DAC(CS) and BECCS, often discussed alongside GGR in public debates, and sometimes even erroneously collated. For these reasons, we included it into our analysis. The second level contains categories for 5 distinct SRM and 11 GGR technologies as well as four categories for general mentions of geoengineering, SRM, GGR, and CCS. Table 1 shows the list of technologies that we included as well as the total number of tweets retrieved for each technology. As tweets can contain keywords from several categories, the sums over subcategories may be larger than the counts at the top level or the total.

**Table 1:** Overview of the classification scheme and composition of the dataset (tweet counts by technology type and average number of retweets, replies and likes per tweet).

**Top-level category**	**Tweet count**	**Technology category**	**Tweet count**	**Retweetspertweet**	**Repliespertweet**	**Likespertweet**
**Geoengineering**	788,668	Geoengineering (general)	788,668	0.80	0.21	1.1

**SRM**	50,644	SRM (general)	36,921	1.26	0.75	3.1
		Stratospheric aerosol injection	7,673	1.00	0.50	1.7
		Cloud brightening	4,166	0.65	0.24	1.4
		Surface albedo modification	1,624	0.61	0.39	1.4
		Cloud thinning	238	0.77	0.31	1.1
		Space shades	744	0.71	0.23	3.3

**GGR**	458,960	GGR (general)	141,237	2.34	0.59	6.8
		Methane removal	3,548	0.80	0.33	1.9
		Ocean fertilization	8,541	0.49	0.14	0.7
		Ocean alkalinization	270	1.67	0.40	3.7
		Enhanced weathering	6,967	0.94	0.35	2.8
		Biochar	19,312	0.65	0.21	1.9
		Afforestation and reforestation	81,518	3.05	0.51	8.5
		Ecosystem restoration	19,808	4.62	0.56	13.1
		Soil carbon sequestration	91,946	1.42	0.27	3.4
		BECCS	20,600	1.49	0.46	3.0
		Blue carbon management	59,286	2.68	0.28	6.8
		Direct air capture	22,959	1.37	0.61	4.7

**CCS**	182,083	CCS	182,083	1.47	0.40	3.3

We collected the data in April 2022. In total, the dataset contains 1,452,184 unique tweets from 314,484 distinct users. Of those, 111,700 tweets (or 7.7%) are retrieved by two or more of the sub-queries. All tweets got 1,917,720 retweets, 460,678 replies and 4,245,861 likes, i.e. on average 1.3 retweets, 0.3 replies and 2.9 likes per tweet. However, the distribution of retweets, replies and likes is highly skewed with the majority of tweets not receiving any reactions at all (i.e. the median is zero). The average number of retweets per tweet is more than twice as high as an estimate for all English-language tweets from the Twitter count API (0.6 retweets per tweet).

### 2.2 Text analysis methods

We augment the corpus-level analysis of our compiled dataset of tweets by applying pre-trained language models. First, we utilize them to create an overview of the topical distribution by embedding all tweets. Second, we classify the sentiments and emotions conveyed in tweets.

To provide a high-level overview of the fine-grained thematic structure of all tweets in our corpus, we embed them into a high-dimensional vector space using BERTweet (Nguyen et al., 2020), which was pre-trained on 850 million English-language tweets posted between 2012 and 2020. For the purpose of visualization and interactive exploration, we reduce the dimensionality using tSNE (Van der Maaten and Hinton, 2008), which preserves the pairwise distances of vectors from the high-dimensional space in a two-dimensional projection. Given the size of our dataset, the projection was trained in batches. This two-dimensional layout enables us to quickly explore and compare entire clusters of tweets based on their prevalent terms and hashtags. Furthermore, we selectively color tweets based on our classification scheme and other meta-information.

To analyze how users perceive the different aspects of geoengineering, we classify all tweets using pre-trained transformer-based classifiers for sentiment. Sentiments are not to be confused with stances: Whereas stance reflects someone’s position towards something, the sentiment indicates whether a thought is expressed in a positive, neutral or negative way. However, anecdotal evidence based on the annotation of a random sample of tweets from our dataset suggests some correlation between the two. Given the large number of available models, we used the models which performed best on commonly used gold-standard datasets. For sentiment analysis, we used two models trained on the dataset shared for the SemEval 2017 Task 4 (Rosenthal et al., 2017) containing 50,000 annotated English-language tweets collected at the end of 2016. Annotations in this dataset are 40% positive and 16% negative sentiment, which could potentially lead to imbalances in the performance to detect both classes.

Emotions differ from sentiments as they do not refer to the general tone of language but rather the presence of expressions associated with specific emotions. For example, a text can contain expressions of fear but can still have a positive sentiment. Whereas sentiment analysis focuses on the tone of a given text, emotion or affect analysis provides a more fine-grained assessment of indicators in the language of a text that point to certain emotions, such as anger, fear, sadness, or disgust. Emotion classification does not actually determine the emotional state of the author at the time of writing, but the emotionality of the used language.

For our analysis, we compared four different emotion classifiers, of which three are pre-trained deep learning models and one is a dictionary approach. There are several taxonomies to define discrete emotions. The SemEval 2018 Task 1 dataset contains 10,000 annotated English tweets with 12 emotion classes (Mohammad et al., 2018), the EmoVent dataset contains 7,303 English tweets with 8 emotion classes (Plaza del Arco et al., 2020), and the GoEmotions dataset contains 58,000 annotated English reddit comments with 27 emotion classes (Demszky et al., 2020). They all suggest their respective mapping to the taxonomy with seven emotion classes proposed by Ekman (1992) or group them into positive, negative, and ambiguous or neutral classes. Aside from deep-learning models trained on these datasets, we also applied a conventional dictionary-based model using the NRC lexicon (Mohammad and Turney, 2013). After predicting the labels, we use the above-mentioned mapping to group emotions into positive (e.g. joy), negative (e.g. fear), neutral (no emotion detected or low model confidence) and thus make results comparable between different classifiers. In the results section, we focus on the findings from the classifier based on the SemEval dataset (Mohammad et al., 2018) given that this one had the highest overlap with our manual annotation (see Section 2.3).

During initial exploration of our dataset, we discovered a large number of tweets containing conspiratorial views, such as the existence of chemtrails, chemwebs, or other secret programs that supposedly seek to control populations with the help of geoengineering. It is important to note that this does not include controversial statements, unconventional technology ideas, or fact-checking a tweet’s content, which is also present in our data set. The strong presence of conspiracy theories in geoengineering debates on Twitter has already been described by Tingley and Wagner (2017) and Debnath et al. (2023). We recorded the presence of potential conspiracies as part of the annotation of a subset of 400 tweets. Using majority voting, we found 35% of tweets to be potentially conspiracy related, with an average Cohen’s kappa score for inter-rater agreement of 0.60.

We use this annotation to develop a filter based on boolean searches of prevalent keywords in these tweets (see Supporting Information). Applying the filter to the entire Twitter dataset identified 17% of tweets as conspiracy-related. We tested the filter with our annotated dataset and found a precision of 94% and a recall of 62%. Thus, the filter can only give a lower estimate of the actual number of conspiracy-related texts.

### 2.3 Evaluation of sentiment and emotion classifiers

To evaluate the performance of the pre-trained sentiment and emotion classifiers, we directly compared their results and also compared the predictions by classifiers to a manually annotated test set. Based on these comparisons, we chose one sentiment and one emotions classifier for the presentation of results in Section 3.

We manually annotated 400 tweets from our dataset to estimate how well the pre-trained classification models, which were trained on different data, can adapt to the domain-specific terminology in our corpus and the potentially distinct phrasing not observed during training. Similar to the inter-rater agreements reported by the publications on the data sets used to train our classifiers that range from 0.10 to 0.38, depending on dataset and emotion class, our five annotators reached a Fleiss’ kappa score of 0.19 for emotions and 0.30 for sentiments. In general, we annotated a larger fraction of our dataset with neutral emotion (63%) than those reported for the training datasets (around 40–50%). However, this corresponds to the predictions of the pre-trained classifiers on our dataset (63.2%).

For each pre-trained classifier, we looked at the distributions of positive and negative sentiments and emotions (Fig. S6). We observe relatively consistent results across sentiment classifiers, both in the share of detected sentiments and their multi-class confusion matrix (see Fig. S2). The second sentiment model that we used (BERTweet trained with SemEval 2017 corpus) produced very similar results to the first one used for the main results of the paper (see Fig. S6). Shares do only differ by a few percentage points. However, sentiment classifier results differ from human annotation on subsets of the data. We coded 53% of the general geoengineering tweets as negative. 13% and 5% of the SRM and GGR tweets received a negative label, respectively. The shares of positive tweets were 5%, 2%, and 11% for general, SRM, and GGR tweets. The remaining tweets were coded as neutral or ambiguous. In comparison, the model classified 35, 25 and 15% as negative as well as 7, 9 and 21% as positive. This comparison shows that the model does not align with shares from manual annotations in each subset of our data but reproduces relative patterns.

The emotion classifiers show a high divergence in shares for the grouped emotions (see Fig. S2 and Fig. S6). The dictionary-based approach (NRC) as well as the RoBERTa model trained on the TweetEval dataset only find fractions of tweets smaller than 40% to be neutral, which neither aligns with the other classifiers, nor our manual annotations. Based on our evaluation, we are most confident in the results of the RoBERTa and BERTweet model trained on the GoEmotions dataset with the reduced Ekman taxonomy of 7 different emotion classes (joy, surprise, sadness, anger, fear, disgust, neutral or other). Our manual annotation recorded diverging shares of negative emotions, ranging from 23% in general geoengineering tweets to 8% in SRM and 1% in GGR tweets. All other tweets were either coded as being neutral or ambiguous, whereas only less than 1% were annotated with a positive emotion. In the paper, we focus on the results of the BERTweet model, because its classifications best align with our manual annotation. Nevertheless, we reviewed results also for the other three classifiers and found mostly consistent patterns in differences between subsets of our data. Although emotion classifiers have low agreement and overall shares differ, the relative patterns mostly align.

Especially for the emotion classification, low inter-coder agreement is a challenging issue. Some of the datasets used to train the classifiers mitigate this problem with multi-label annotation (i.e. allowing to assign more than one emotion during annotation). However, this can lead to contradictory annotation (e.g. neutral plus a non-neutral emotion) and may distort classifiers towards detecting more emotions than a single annotator would identify. This may explain the low shares of predicted neutral labels, which are not in line with our manual annotation. We therefore suggest developing better internal and external evaluation approaches of emotion analyses on tweets than often seen in the literature (Loureiro and Alló, 2020, Qiao and Williams, 2022).

## 3 Results

### 3.1 Attention and user engagement

**Fig. 1:**
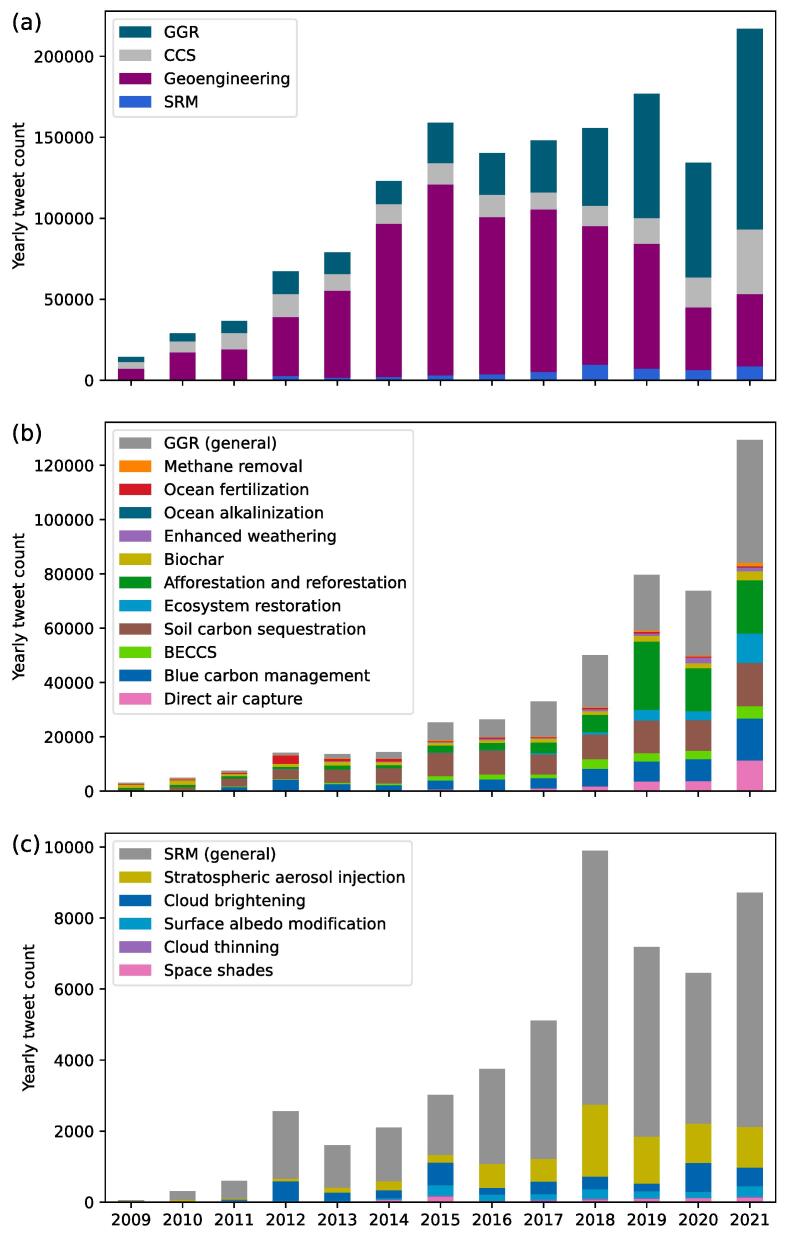
Yearly tweet counts. (a) For general geoengineering (Geoengineering), greenhouse gas removal (GGR), solar radiation management (SRM) and carbon capture and storage (CCS). (b) GGR tweet counts by technology (breakdown of green bar in panel (a)). (c) SRM tweet counts by technology (breakdown of blue bar in panel (a)). Data for years 2006 to 2008 are not shown because the counts for these years are zero or very low.

Attention on Twitter focuses on general geoengineering and GGR. We measure the (relative) attention to technologies and topics based on the number of tweets containing particular keywords. Whereas the attention to GGR and geoengineering is very high, the share of tweets on SRM is only 3.4% in our dataset. Tweets containing general geoengineering terms make up about half of the entire dataset (54%), followed by GGR (35%) and CCS (13%), but there is a distinct temporal dynamic towards the latter (see Fig. 1).

Attention on Twitter shifted from general geoengineering to GGR. Fig. 1a shows that the development over time can be divided into distinct phases: First, the increase up to 2013 coincided with the increase of the total number of English-language tweets on Twitter (see Fig. S5) and is therefore mostly driven by the overall expansion of the social media platform. Our estimate of the total number of English-language tweets over time suggests that this number peaked around 2013 after its initial rapid growth and decayed slowly until 2018, when growth picked up again until today. Second, geoengineering topics expanded further between 2013 and 2015. Based on the aforementioned estimates, the ratio of geoengineering tweets more than tripled (from one in every 271,000 before 2014 to one in every 75,000 after). The number of tweets using general geoengineering keywords peaked in 2015 and has decreased since then. Third, this decrease has been compensated by a strong expansion of tweets on GGR and related technologies in recent years. Since 2020, the share of GGR tweets is even higher than the share of general geoengineering tweets (see Fig. S7a). The increase was only interrupted by a drop in 2020 following the outbreak of the Covid-19 pandemic. However, attention quickly recovered in 2021 and was about 75% higher than in 2020 and 62% higher than in 2019, the year with most attention before the pandemic. CCS saw a similar increase in 2021, but has received more stable attention in the years between 2011 and 2018. Communication about SRM grew slowly until a peak in 2018, but remains at very low levels compared to GGR and general geoengineering discussions.

Attention to specific GGR methods grows, while attention to SRM remains low and at a general level. The number of overall tweets on GGR is not only more than an order of magnitude larger, but there are also many more references to specific GGR methods, as indicated by the number of tweets with technology-specific keywords. Of the more than 450,000 tweets on GGR, about 31% contain generic keywords such as “greenhouse gas removal”, “carbon dioxide removal”, or “negative emissions”. The remainder comprises technology-specific communication on soil carbon sequestration (20%), afforestation/reforestation (17%), and blue carbon (12%). Direct air capture, bioenergy with CCS, ecosystem restoration, biochar, ocean fertilization and enhanced weathering each have shares of 5% or less. Methane removal, the only method for greenhouse gases other than carbon dioxide in our data set, and ocean alkalinization are the least discussed GGR methods, with only about 3,500 and 270 tweets, respectively. In contrast, of the about 57,000 tweets on SRM, more than half contain general keywords (66%) and only a small number of tweets use technology-specific keywords such as stratospheric aerosol injection (22%). All other SRM technologies have shares of less than 10% of the total number of SRM related tweets.

Users engage more with GGR topics than with SRM and general geoengineering. On average, general geoengineering tweets were retweeted around 0.8 times and received 1.1 likes per tweet. We see slightly higher engagement in SRM (and CCS) tweets with 1.2 (1.5) retweets and 2.7 (3.3) likes per tweet and much higher engagement in GGR tweets with 2.1 retweets and 6.0 likes per tweet. These differences are not driven by tweets from users that tweet very frequently about these topics, as the analysis of average retweets, replies and likes per user highlights. Average per user retweets are 0.4, 1.0 and 1.4 for general geoengineering, SRM and GGR, respectively (see Table S1 for further details). The distributions of retweets and likes per tweet as well as per user are highly skewed, with a majority of tweets not receiving any reactions, which gives median values of zero for each of these distributions.

The distribution of tweets per user is also strongly skewed (see Fig. S4) and follows a power-law as other social and online phenomena (Newman, 2005). While on average, there are 4.6 tweets per user in our dataset, the 1% most active users posted about 44% of the tweets in our dataset. The next 9% contributed 26% of all tweets; 63% of all users tweeted only once and thereby contributed 13% of all tweets. These numbers also differ between subsets: Average tweets per user are higher for general geoengineering tweets (5.8) and lower for SRM, GGR and CCS (2.3, 2.7 and 3.3, respectively). Also the share of tweets from the top 10% most active users differs considerably between 76% for general geoengineering tweets, 60% for CCS, 56% for GGR and 54% for SRM. These differences suggest that Twitter communication about general geoengineering is more concentrated on a few users than for CCS, GGR and SRM.

### 3.2 Sentiments and emotions

**Fig. 2:**
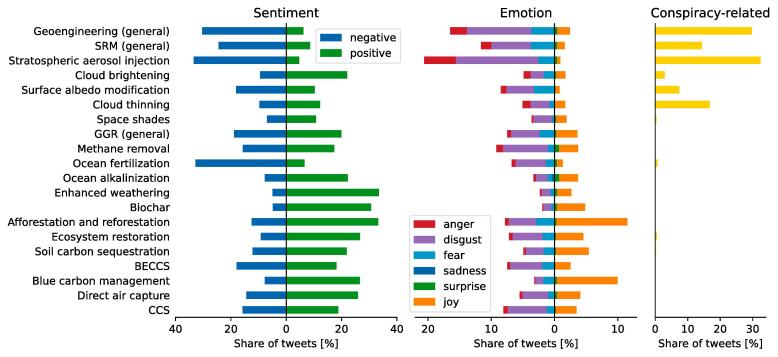
Share of sentiments and emotions as well as share of conspiracy-related tweets for each technology category.

We classify tweets with respect to their sentiment, or tone, as described in Section 2.2. Fig. 2 compares the shares of tweets with positive and negative sentiments in subsets of our dataset. These shares are computed at the level of tweets, but looking at average sentiments grouped by user yields very similar results (see Fig. S8). General geoengineering tweets have high shares of negative sentiment (30%) and low shares of positive sentiment (6%). Because this makes up more than half of the dataset, this leads to a high overall share of 23% negative tweets compared to only 13% positive ones in the entire dataset. The share of negative GGR tweets is much lower (14%) and the share of positive ones higher (24%), also compared to SRM (24% negative, 9% positive). CCS tweets are also more positive (19%) than negative (15%).

We observe that technology-specific subsets of tweets have on average lower shares of negative sentiment than tweets containing general keywords on geoengineering, SRM or GGR. Interestingly, stratospheric aerosol injection for SRM and ocean fertilization for GGR are the only technologies that feature a higher share of negative sentiments than general tweets. This is related to concerns that have led to public opposition towards field experiments on those technologies, such as detrimental environmental effects, slippery slope towards large-scale implementation, and poor governance or even illegality of projects (Low et al., 2022, Strong et al., 2009, Osaka, 2023).

Sentiments are more positive for technologies perceived closer to nature. Across GGR and SRM technologies there are substantial differences in the tone of respective tweets. Our results highlight that tweets on GGR technologies tend to be associated with more positive than negative sentiments, except ocean fertilization. BECCS has broadly balanced positive and negative sentiments. Land-based biological GGR methods such as afforestation, biochar, blue carbon or ecosystem restoration have high shares of tweets with positive and low shares of tweets with negative sentiments. Among the SRM technologies, stratospheric aerosol injection has the lowest share of positive vis-a-vis the highest share of negative tweets. The pattern is reversed for cloud brightening and also for space-based sunshields, but tweets counts are too low for a reliable signal. All other SRM technologies have rather low shares of both positive and negative tweets.

We analyze potential framing effects by comparing the share of sentiments between GGR tweets that additionally mention general geoengineering keywords to those that do not. Tweets containing both GGR and general geoengineering keywords are composed of 30% negative, 64% neutral, and 6% positive tweets. GGR tweets without reference to geoengineering, conversely, contain 14% negative, 63% neutral, 23% positive sentiments. This shows that framing GGR as a geoengineering technology leads to less positive and more negative sentiments.

Emotions follow similar patterns to sentiments and add nuance to the findings from the sentiment analysis. For the entire dataset, we find 3% positive, 85% neutral and 12% negative emotions. Fig. 2 shows shares for all technology classes. We can see patterns similar to the sentiments: Tweets with more general keywords tend to entail more negative emotions, and GGR tweets are associated much more with positive emotions than SRM and General tweets. Compared to sentiment analysis, both positive and negative emotions are less prevalent. Other notable differences to the patterns observed for sentiments include less negative emotions towards ocean fertilization and more positive emotions towards blue carbon.

We find that the specific emotion ‘disgust’ dominates in both general geoengineering and SRM tweets (10% and 7% respectively, see Fig. 2). This is followed by ‘fear’ and ‘anger’ (2–3.5%), while ‘joy’ only contributes 2% and 1%, respectively. For GGR, the picture is different: ‘joy’ (6%) is followed by ‘disgust’ (4%), and ‘fear’ (2%), while anger is below 1%. ‘Sadness’ and ‘surprise’ are hardly present in any of the subsets of tweets. For specific technologies, the relatively high shares of ’joy’ for afforestation/reforestation (11%) and blue carbon (10%) and the high share of ’disgust’ for stratospheric aerosol injection (13%) are notable. There are also considerable differences between tweets that can be related to conspiracy theories and others: the former have a 2.9 times higher share of ‘anger’ and 1.8 times higher share of ‘disgust’ while only half the share of ‘joy’ than the latter.

### 3.3 Conspiracies and semantic proximity

High shares of general geoengineering and SRM tweets contain conspiracy-related keywords. Using a filter based on a boolean keyword search, we identify a lower estimate for the share of tweets related to conspiracy theories in each subset of our data (Fig. 2). We find conspiracy theories to be most prevalent for general geoengineering tweets (30%). For SRM tweets, the share is still high at 16%, while GGR and CCS tweets scarcely contain conspiracy-related keywords (0.15% and 0.07%). Among technology-specific tweets, the share of conspiracy-related tweets is highest for stratospheric aerosol injection with 32%. A prominent example of these conspiracies is the “chemtrails” theory, whose adherents believe that nefarious actors (often governments or secret societies) are injecting chemicals into the atmosphere, disguised as contrails of airplanes. Nonetheless, it is likely that these numbers underestimate the amount of conspiracy-related tweets in our dataset, as they capture only about two thirds of manually annotated tweets.

Over time, we find that the share of conspiracy theory-related tweets varies on a monthly basis between 20% and 50% for general geoengineering tweets. For both general and SRM tweets, there is a trend towards lower shares of conspiracy-related tweets starting around 2016. While the share of tweets on GGR with conspiracy-related content is very low, those tweets containing both GGR and general geoengineering keywords have a much higher share of 1%, which can be linked to conspiracies. Interestingly, conspiracy-related tweets receive far less likes (1.1 compared to 3.3 likes per tweet) and are retweeted slightly less than other tweets (1.2 compared to 1.3 retweets/tweet).

**Fig. 3:**
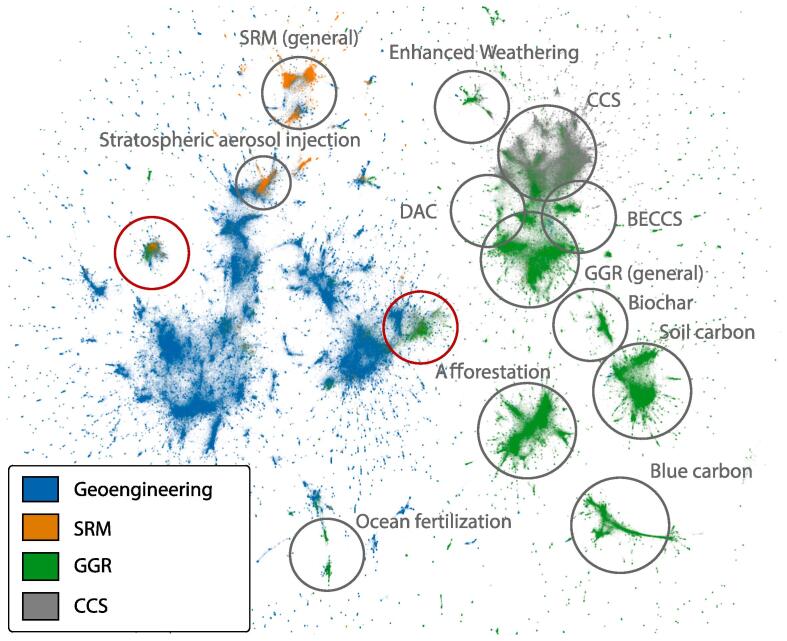
Map of tweets using a two-dimensional projection of the high-dimensional tweet embeddings. Each dot in the figure corresponds to a tweet and is colored according to top-level categories (blue: General geoengineering, orange: SRM, green: GGR, grey: CCS). The relative distance between dots shows their semantic similarity. Manual annotations (circles) point to regions in which tweets from a technology category are concentrated (based on Fig. S1).

As described in Section 2.2, we embed all tweets in a high-dimensional space using a state-of-the-art natural language model (Nguyen et al., 2020). Fig. 3 shows the result of reducing this space to a plottable two-dimensional map. Each dot in the map represents one tweet. Close pairwise proximity between dots indicates higher semantic similarity in the content of tweets. The locations of tweets in the embedding space strongly correlate with the technology category, indicating that the queries produce coherent and largely consistent sets of tweets.

In the map, most clusters with SRM tweets also contain general geoengineering tweets. This points towards SRM being more often mentioned in similar contexts as geoengineering, using similar frames. Most GGR and CCS tweets are well separated from general geoengineering in the map. Exceptions are two clusters containing both geoengineering and GGR tweets (marked as red circles in Fig. 3). Both contain high shares of conspiracy-related tweets (13 and 7%). The map also shows that similar technologies, like CCS, BECCS and DAC, are located closer to one another than more diverse technologies such as soil carbon, afforestation/reforestation and enhanced weathering.

## 4 Discussion

### 4.1 Trends and drivers of perceptions

Our findings regarding the attention and user engagement with different geoengineering technologies are in line with evidence from the literature. The low share of tweets on SRM points towards little familiarity with these types of technologies. This aligns with the finding from surveys that these technologies are hardly known in the public (Mercer et al., 2011, Visschers et al., 2017). The greater attention to and more technology-focused discussions on GGR suggest greater awareness of and familiarity with GGR. This is particularly apparent for conventional and land-based GGR methods such as afforestation or soil carbon management as also found in surveys (Jobin and Siegrist, 2020, Sweet et al., 2021). This might also explain why users are much more likely to retweet and like tweets about GGR. Another explanation for sharing GGR more than other geoengineering tweets could be that these technologies have much higher support rates in surveys (Jobin and Siegrist, 2020). Apart from this, GGR is moving up on the policy agenda in many industrialized countries as net-zero goals both at the level of countries and firms are announced (Schenuit et al., 2021, Boettcher et al., 2023) and is pushed by start-ups, engineers and scientists who disseminate content on these technologies. These factors also contribute to increasing trends and higher sharing metrics on Twitter.

Our result that GGR is more positively discussed than SRM and geoengineering in general is also well in line with the finding from surveys that SRM technologies have lower support rates than GGR (Jobin and Siegrist, 2020, Carlisle et al., 2020). Studies report higher support for GGR methods like afforestation/reforestion, biochar and soil carbon management (Sweet et al., 2021, Wolske et al., 2019, Bellamy, 2022), for which we also find higher shares of positive sentiments and emotions. Survey studies explain this by the perceived naturalness of these methods (Sweet et al., 2021, Shrum et al., 2020, Bertram and Merk, 2020). The mostly negative sentiments in SRM tweets are further driven by controversial debates both in academia and in the public about these technologies, their unknown side-effects, and their highly uncertain implications for governance (Macnaghten and Szerszynski, 2013, Aldy et al., 2021).

Furthermore, we find that general geoengineering as well as SRM tweets have a much higher share of conspiracy-related content. Surveys have found that a high percentage of a representative US population tends to believe that there is some truth in the ‘chemtrails’ conspiracy theory (Mercer et al., 2011, find that 3% believe it is true, 14% believe it is somehow true). The number of conspiracy-related tweets, and users sharing these tweets, is comparatively larger than shares from this survey, but this is not surprising as Twitter is known to over-represent extreme positions. While strong links between geoengineering, SRM and conspiracy theories on social media have been found previously (Tingley and Wagner, 2017, Debnath et al., 2023), our comparative approach shows differences between SRM and geoengineering tweets and that this finding does not extend to GGR debates. Finally, we find a decreasing trend in the share of conspiracy-related content in our dataset, which might be the result of Twitter’s (then) increased effort to reduce misinformation on its platform (Allcott et al., 2019) and spreaders of conspiracy theories moving to other topics such as Covid-19 and anti-vaccination (Shahi et al., 2021).

### 4.2 Geoengineering framing

Our analysis of tweets, how their content is related in the semantic map and the sentiment and emotion in different subsets indicate that using a geoengineering framing results in more negative sentiment and may be more prone to misinformation. The semantic map in Fig. 3 shows that SRM has a much higher semantic proximity to general geoengineering tweets. This may be an indication for SRM often being framed in the context of geoengineering, while this is the exception for GGR. One such exception is the closer semantic proximity of ocean fertilization tweets to geoengineering, which might be linked to the controversial experiments discussed for this technology (Low et al., 2022).

The semantic proximity of tweets about geoengineering (general), SRM (general), stratospheric aerosol injection, and to a lesser extent ocean iron fertilization in Fig. 3 suggests that “geoengineering” might be thought by many to refer specifically to these options, and to exclude most forms of GGR. Such an interpretation might also be linked to the very similar sentiments, emotions, and tendency for conspiracy theories in these technologies.

Last but not least, the notion “geoengineering” is used both to describe a group of technologies (with contested system boundaries) as well as to ascribe certain properties to one or many technologies, often in a pejorative way. This points to “geoengineering” being a floating signifier, i.e. a concept with no commonly agreed upon meaning (Farkas and Schou, 2018). As the “geoengineering” concept is comparably new, not only for the public, but also in academic discussions (Keith, 2000), people attach very different meanings to it, making it an ideal term for political contestation. This has implications for what terminology is most effective for communicating with the public about different options of emerging climate technologies for mitigating and reducing the impact of climate change. In particular, it suggests that using the term “geoengineering” to refer to GGR options negatively associates them with much more controversial SRM technologies.

### 4.3 Policy implications

Our results highlight stark differences in how SRM and GGR are communicated on Twitter. There is little discussion of SRM and it is overall negative and often related to conspiracies, which points to a premature public debate. On the contrary, GGR is much more discussed, hardly linked to conspiracies and mentioned more often in positive contexts – with the exception of ocean fertilization. This points to much more support for GGR methods than SRM, as also found in the survey-based literature (Jobin and Siegrist, 2020, Carlisle et al., 2020). We therefore recommend to clearly distinguish between SRM and GGR in policy debates and avoid lumping them together under the umbrella term “geoengineering”. Public support for large-scale deployment of GGR is important for keeping the Paris climate goal within reach. To secure this support for GGR, communicators should avoid the “geoengineering” framing.

It may also be advisable to minimize the joint discussion of SRM and GGR policies and implementation. Discussing these very different approaches jointly comes with the risk of “controversy spillovers” from more problematic SRM to less problematic GGR (Cuppen et al., 2020, Westlake et al., 2023). Such spillovers also influence the perception of single GGR technologies. For example, BECCS and DACCS might be negatively impacted by scepticism about CCS from fossil sources. Methods such as afforestation, blue carbon management and soil carbon sequestration (sometimes labelled “nature-based solutions”) have highest approval in surveys and are therefore likely the least controversial to implement in the near future.

More broadly, computer-assisted social media analyses can provide a preview and overview of potential concerns, opposition and appraisal surfacing in the public discussion about the development and deployment of emerging technologies. This can help policy makers and other stakeholders to address such concerns early on. Monitoring these debates can also inform the development of strategies to counter and inoculate the public against misinformation. Our study has shown that part of the debate, particularly about geoengineering and SRM, are shaped by emotions and disinformation. Disinformation campaigns often appeal to user’s emotions rather than engaging rationally in a debate because emotional engagement increases belief in and thus sharing of misinformation (Martel et al., 2020, Horner et al., 2021). This can lead to entire social media debates being dominated by minority opinions, which might spread further. In times of populist opposition to climate solutions, it is therefore important to consider these non-rational elements in policy discourses when communicating about new technologies and policies. Given the plasticity of perceptions of future technologies and taking the limitations of social media data into account (see next section), further survey-based and qualitative social science work is needed to deepen insights into how public perceptions are formed and which factors influence support for emerging climate technologies.

### 4.4 Limitations

Our findings are based on Twitter data, which is known for specific user characteristics, communication patterns as well as algorithmic feedback mechanisms (Sen et al., 2021, Mellon and Prosser, 2017). Some effects of these biases on our results are reduced by the comparative approach we chose. For example, we can assume that the influence of demographic characteristics of users on posting behavior is similar across subsets of our data, such that differences between subsets are not driven by them. Additionally, our keyword-based searches come with some caveats. First, we only include those tweets in our analysis that explicitly mention keywords related to geoengineering or SRM and GGR technologies. However, some tweets may be part of longer debates between users or threads that focus on these topics without mentioning these specific keywords in each tweet. Second, despite the great care we took in designing the search queries (see Section 2.1), we might still miss relevant keywords that our multidisciplinary author team was not aware of, thereby unintentionally introducing biases into the dataset. The above comparison of our main findings with results from representative surveys indicates, however, that the Twitter user base posting about geoengineering seems to express qualitatively similar attitudes. This gives us confidence in the robustness of our findings.

Our comparison of several state-of-the-art sentiment and emotion classifiers revealed a huge disparity of results between emotion classifiers, while the labels of sentiment classifiers had a much higher agreement. The evaluation of emotion classifiers in our domain was complicated by two factors: First, emotion classifiers use different labeling schemes that are not easily comparable and cannot be matched to each other. This is why we mapped results to three classes of positive, negative and neutral emotions for the comparison between classifiers. Secondly, manually annotating the emotion of short texts such as tweets can be ambiguous because there are often only slight indications of emotional language features. For example, mentioning risks of an SRM technology like reducing crop yields can be interpreted as an expression of fear, but could also simply be neutral information. We therefore expect that automated sentiment and emotion classification also involves mistakes. This can affect the absolute number of annotations for each category. However, we assume that errors in automatic annotation are evenly distributed across categories and that therefore the reported shares, i.e. the relative numbers, of each category of sentiments and emotions are much more reliable than single annotations. Comparing the results of the emotion and sentiment analysis confirms that this assumption is reasonable. Our results highlight that some of the identified sentiment is driven by emotional wording, with negative emotions being more prevalent than positive ones even in subsets with balanced sentiments. Thus, the emotion analysis can be used to validate patterns found with sentiment analysis and add more nuance to the ways that particular technologies are associated with positive or negative sentiment. However, we caution against overinterpreting the results of the emotion analysis because even manual annotation of tweets comes with low agreement between different annotators and the agreement between majority annotation with the emotion classifier is low.

## 5 Conclusions

This paper provides a comprehensive overview of communication about geoengineering and related technologies on Twitter over the entire history of the social media platform. It expands previous analyses both in scope—looking at SRM and GGR—and through the use of a comparative research design. As such, it presents a complementary line of evidence on public perceptions of emerging technologies, which are constantly shaped by public debates and are therefore hard to assess by traditional survey methods.

Attention on Twitter has shifted from general geoengineering communication to specific GGR technologies. The term “geoengineering” often comes with negative connotations as do many SRM technologies. In contrast, most GGR technologies are discussed positively at large, especially those using natural sinks such as afforestation, ecosystem restoration, blue carbon and soil carbon sequestration. This confirms findings in the survey-based literature: methods that are perceived as more natural receive more approval. Our results suggest that SRM is strongly linked to the geoengineering framing, while general GGR and most GGR methods are increasingly discussed independently. In parallel, GGR methods that have been discussed for much longer in other contexts – such as forest management, biochar, ecosystem restoration – are now being incorporated into climate solution debates and repurposed as carbon removal, which can be both a challenge and opportunity for their public support. These findings imply that policy debates about GGR should avoid the “geoengineering” frame and discuss GGR separately from SRM to circumvent controversy spillovers.

The work presented in this paper is a first step towards a better understanding of public perceptions about the emerging technologies of SRM and GGR on social media. Overall, we contribute to the growing efforts to systematically collect and analyze data pertaining to public opinion about climate change on social media, and factors that influence it (Kirilenko et al., 2015, Moore et al., 2019, Effrosynidis et al., 2022). To extend insights into public perceptions of these topics, future work should characterize user groups and networks between users to learn about the different engagement of stakeholders such as politicians, journalists, scientists or business representatives. Furthermore, links to external content in geoengineering tweets provide rich materials about what types of information sources people use and share. Finally, extensions to other social media such as Reddit could help to generalize findings across platforms.

## CRediT authorship contribution statement

**Finn Müller-Hansen:** Methodology, Visualization, Writing - original draft, Formal analysis, Data curation, Validation, Conceptualization, Writing - review & editing. **Tim Repke:** Formal analysis, Data curation, Validation, Conceptualization, Writing - review & editing. **Chad M. Baum:** Conceptualization, Writing - review & editing. **Elina Brutschin:** Conceptualization, Writing - review & editing. **Max W. Callaghan:** Conceptualization, Writing - review & editing. **Ramit Debnath:** Conceptualization, Writing - review & editing. **William F. Lamb:** Conceptualization, Writing - review & editing. **Sean Low:** Conceptualization, Writing - review & editing. **Sarah Lück:** Conceptualization, Writing - review & editing. **Cameron Roberts:** Conceptualization, Writing - review & editing. **Benjamin K. Sovacool:** Conceptualization, Writing - review & editing, Funding acquisition, Supervision. **Jan C. Minx:** Funding acquisition, Supervision, Conceptualization, Methodology, Writing - review & editing.

## Declaration of Competing Interest

The authors declare that they have no known competing financial interests or personal relationships that could have appeared to influence the work reported in this paper.

## Data Availability

As per Twitter’s Terms of Service, sharing the full tweet data is not feasible. However, to allow for reproducibility, we provide the search queries in the supplementary material and the ids of retrieved tweets with categorizations as well as our code in an archive on Zenodo at https://doi.org/10.5281/zenodo.10008167.

## Footnotes

1 At the time of revising this paper (Sept. 2023), Twitter has been renamed to X. However, we use the platform’s old name in the paper because this was its original name at the time of writing and data acquisition.
