# Supplementary material for: Attention, sentiments and emotions towards emerging climate technologies on Twitter
Source: Glob Environ Change. 2023 Dec;83:102765. doi: 10.1016/j.gloenvcha.2023.102765 (PMC10730943; doi:10.1016/j.gloenvcha.2023.102765)
Supplement: Supplementary data 1 [file mmc1.pdf]

Supporting Information

This document contains supplementary figures, tables and method descriptions for the paper "Attention, sentiments and emotions towards emerging climate technologies on Twitter" by Müller-Hansen et al. (Global Environmental Change, 2023). Apart from this document, data and code for reproducing results are available on Zenodo at `https://doi.org/10.5281/zenodo.10008167`.

S1 Supplementary tables and figures

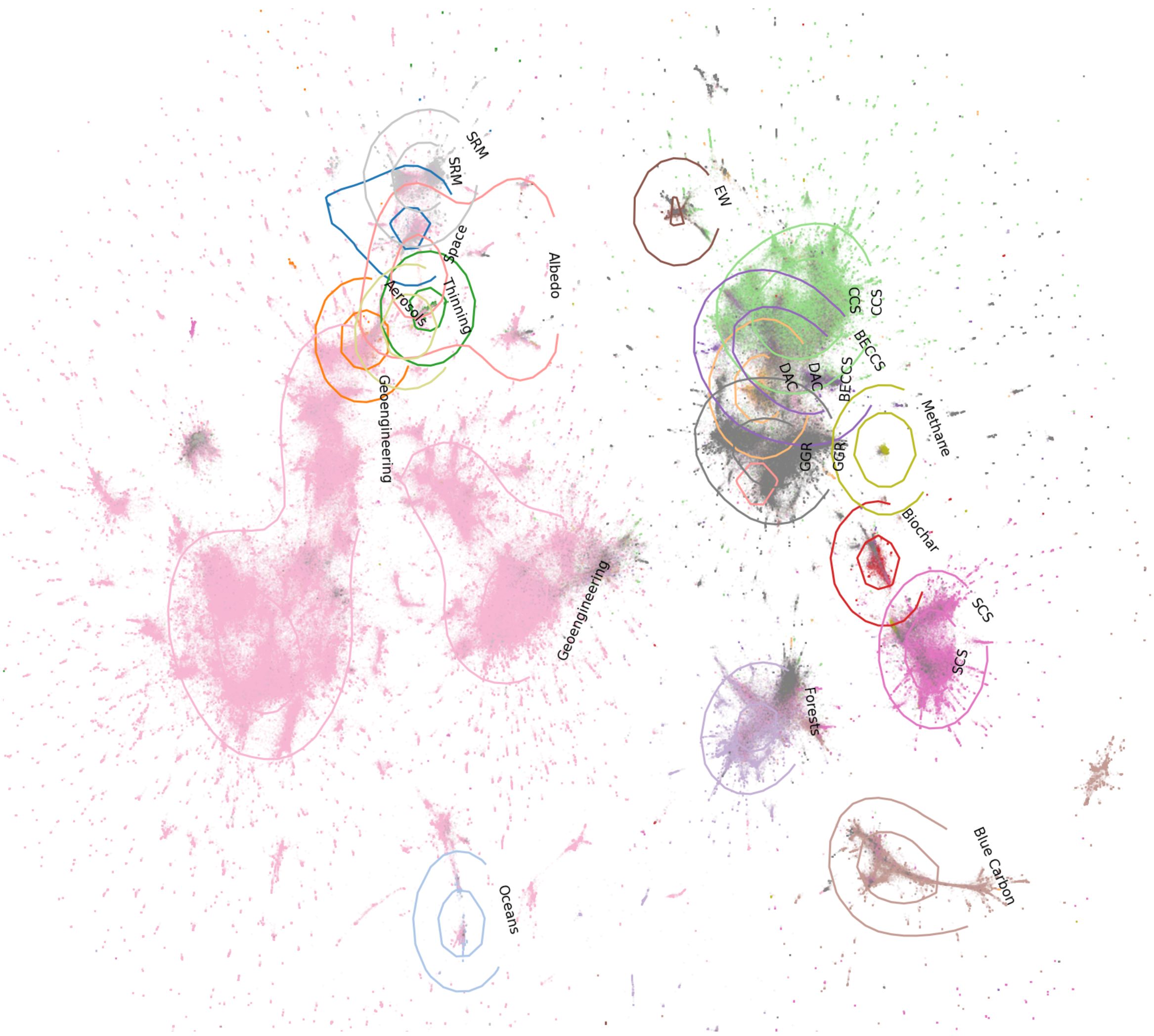

Figure S1: Map of tweets using a two-dimensional projection of embedded tweets, colored by technology category. Automatically generated labels indicate regions of high concentrations of tweets from technology categories.

Table S1: Average number of retweets, replies and likes per user.

**Technology category**	**Avg. retweets / user**	**Avg. replies/ user**	**Avg. likes/ user**
Geoengineering (general)	0.41	0.19	1.07
SRM (general)	1.06	0.66	3.50
Stratospheric aerosol injection	0.86	0.53	1.75
Cloud brightening	0.52	0.19	1.23
Surface albedo modification	0.70	0.41	1.77
Cloud thinning	0.52	0.23	0.88
Space shades	0.83	0.29	4.37
GGR (general)	1.39	0.41	4.77
Methane removal	0.81	0.35	1.93
Ocean fertilization	0.44	0.13	0.69
Ocean alkalinization	1.58	0.34	3.49
Enhanced weathering	1.01	0.36	3.14
Biochar	0.60	0.20	1.75
Afforestation and reforestation	2.16	0.46	7.00
Ecosystem restoration	1.87	0.34	5.68
Soil carbon sequestration	1.20	0.28	3.48
BECCS	1.06	0.31	2.52
Blue carbon management	1.78	0.26	5.36
Direct air capture	1.00	0.46	3.93
CCS	1.02	0.32	2.70

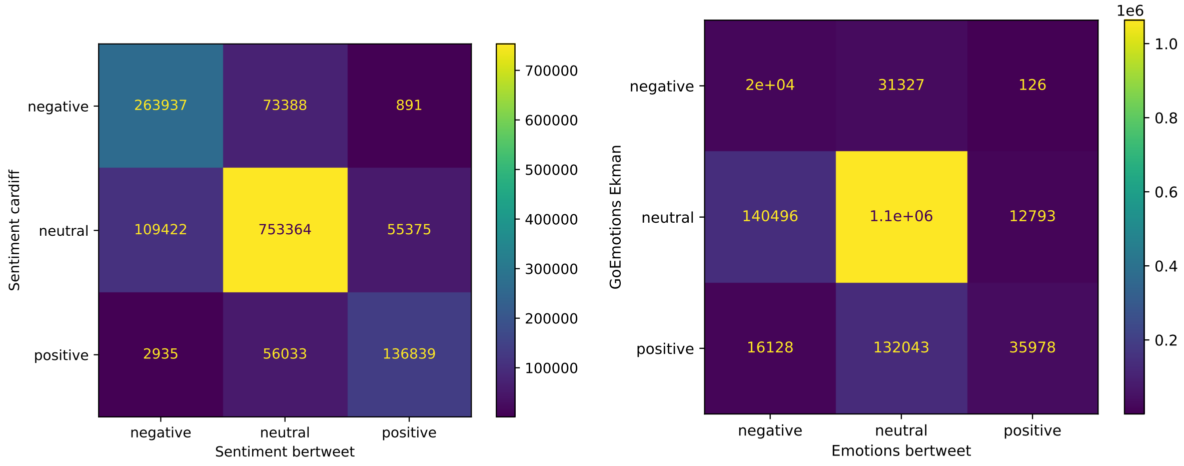

Figure S2: Comparison matrix between labels from the two sentiment classifiers (left panel) and the two best performing emotion classifiers (left panel). Note that we mapped emotions from the scheme of each classifier to {positive, neutral, negative} to make the very different schemes comparable.

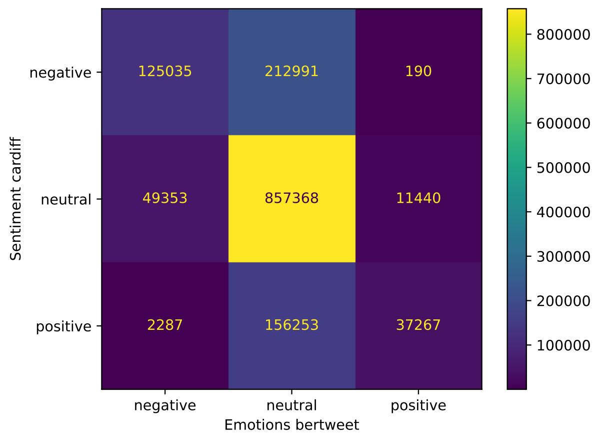

Figure S3: Comparison matrix between the selected sentiment classifier and the selected emotions classifier.

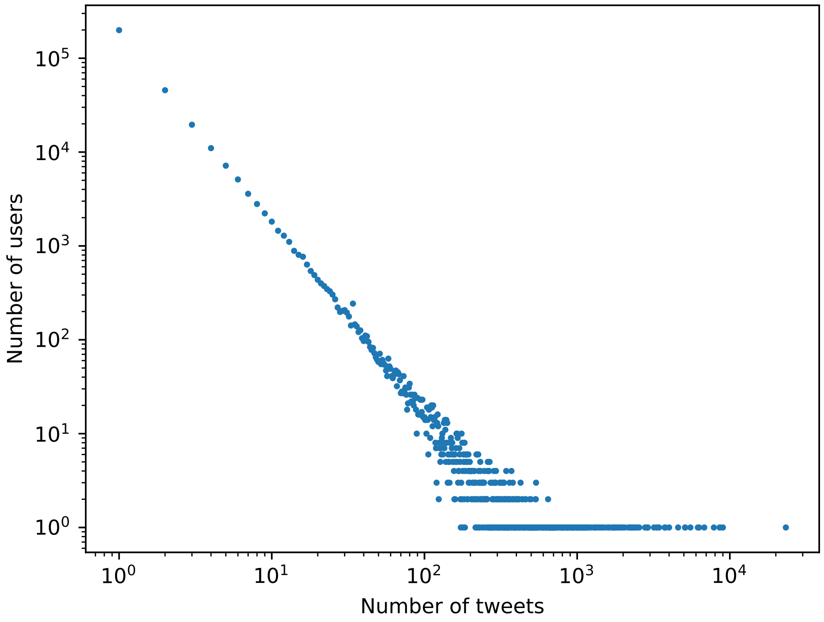

Figure S4: Histogram showing the number of users that posted a certain number of tweets.

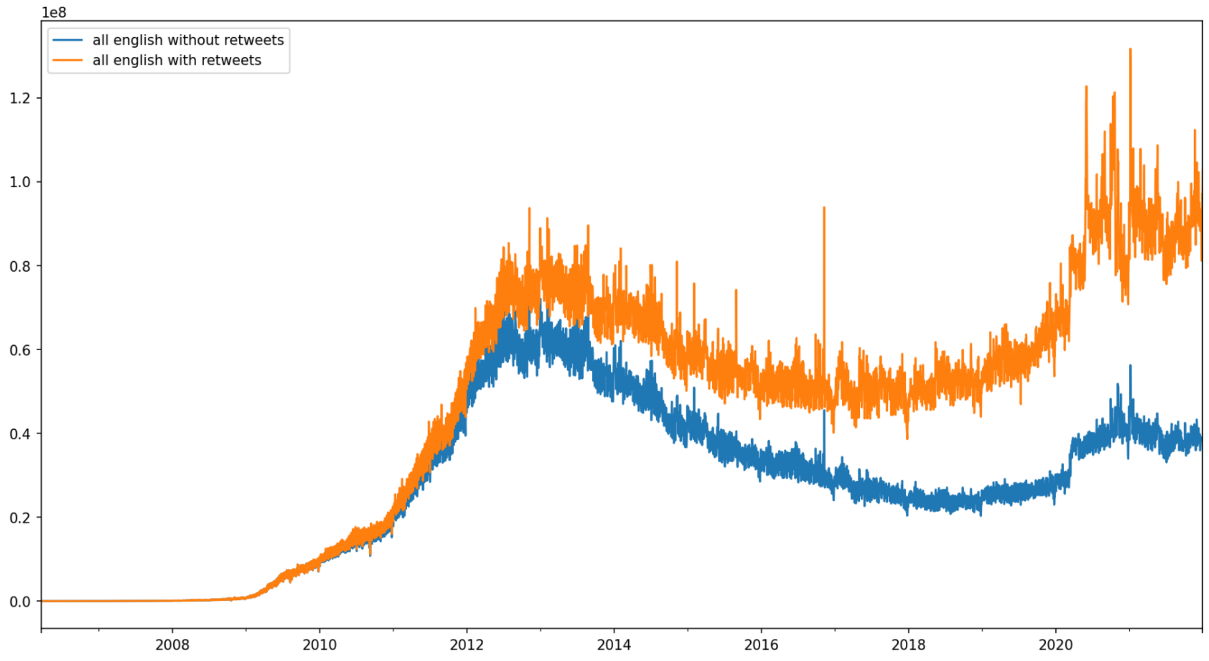

Figure S5: Number of English-language tweets over time. The numbers have been estimated by searching for the 100 most common English words in tweets via the Twitter count API (`https://api.twitter.com/2/tweets/counts/all`).

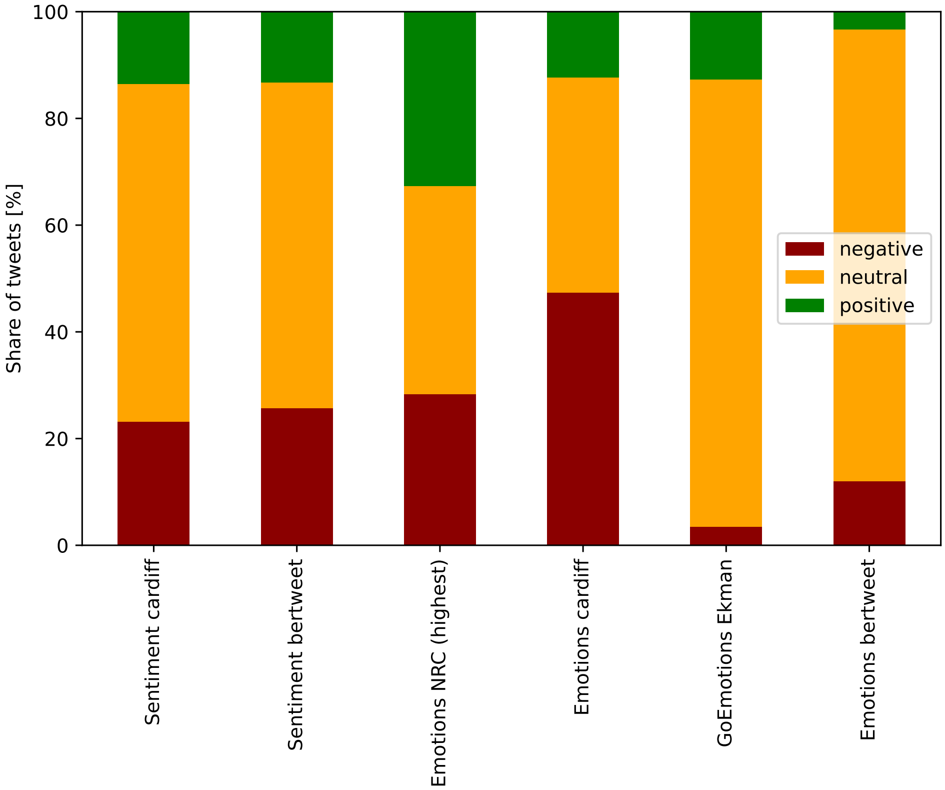

Figure S6: Comparison of labels from different sentiment and emotion classifiers. Note that we mapped emotions from the scheme of each classifier to {positive, neutral, negative} to make the very different schemes comparable.

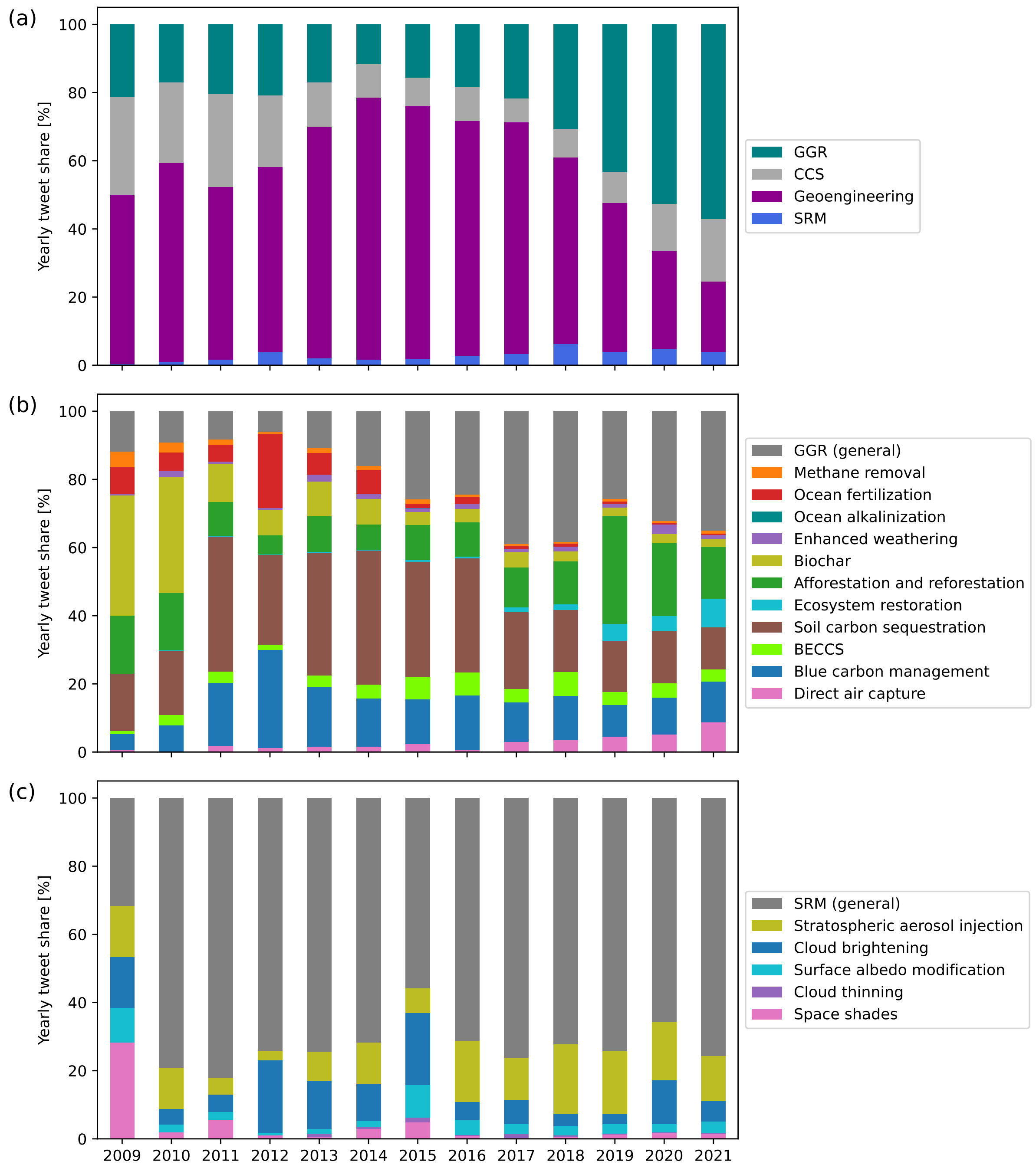

Figure S7: Yearly share of tweets of (a) Level 1 categories in the entire dataset, (b) level 2 categories in GGR tweets and (c) SRM tweets.

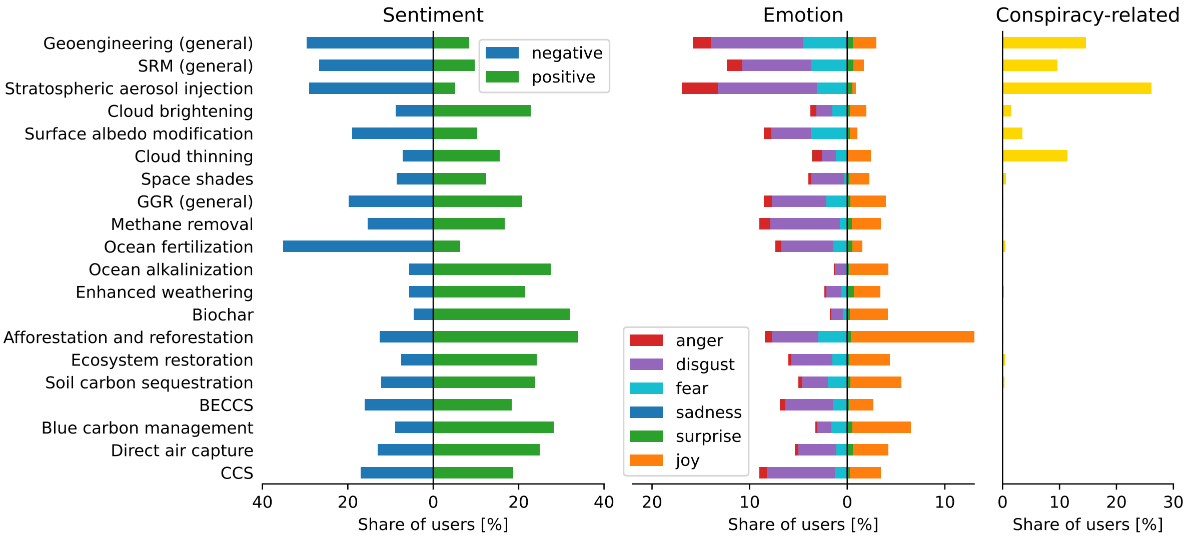

Figure S8: Share of sentiments and emotions and share of conspiracy-related tweets for each technology category by user. To compute this metric, we first took the average sentiment of all tweets by each user and then averaged over all users. This is equivalent to a weighted average over tweets with a weight of one over the number of tweets by the user posting the tweet.

S2 List of subqueries for data gathering

The following list of keyword searches (Table S2) is based on queries developed for identifying scientific literature on carbon dioxide removal techniques Minx et al., 2018. Furthermore, we use other reviews on the topics of geoengineering, solar radiation management and greenhouse gas removal to extend the list of relevant keywords Cummings et al., 2017; Vaughan and Lenton, 2011; Caldeira et al., 2013. These keyword collections were adapted to return high shares of tweets relevant to the covered topics. We iteratively screened random samples of the results for each subquery to ensure that tweets are relevant to the covered topics. During this process, we dropped some subqueries that yielded mostly irrelevant results or modified the queries to include climate-related or exclude keywords not related to climate.

During the refinement of queries, we made an effort to make sure that we do not miss large parts of tweets on geoengineering-related topics. This is why we extended some of subqueries for techniques that initially returned only few results, which resulted in some imbalances in the number of subqueries per technique.

Table S2: Table of subqueries for data gathering, count of unique tweets and explanation.

category	technology	subquery	count	explanation
Geoeng.	Geoengineering (general)	geoengineering -"solar geoengineering"	666176	Tweets mentioning "geoengineering" but not "solar geoengineering"
Geoeng.	Geoengineering (general)	"climate engineering"	53325	"Climate engineering" is often used as a synonym for "geoengineering". Other synonyms like "planetary engineering" did mostly not cover geoengineering topics.
Geoeng.	Geoengineering (general)	geo-engineering	53214	Common alternative spelling for "geoengineering"
Geoeng.	Geoengineering (general)	"geo engineering"	41421	Common alternative spelling for "geoengineering"
SRM	SRM (general)	"solar radiation management"	12011	"Solar radiation management" refers to technologies that modify the radiation balance of incoming and reflected sunlight.

Continued on next page

category	technology	subquery	count	explanation
SRM	SRM (general)	”solar geoengineering”	20873	Synonym for SRM
SRM	SRM (general)	”sun dimming”	4290	A lot of communication about SRM uses phrases like ”sun dimming” to describe those technologies.
SRM	SRM (general)	solar ”climate intervention”	200	
SRM	SRM (general)	”earth radiation management”	20	Synonym for SRM
SRM	aerosol injection	”aerosol injection”	6332	”Stratospheric aerosol injection” is the most discussed SRM technique. As many tweets use ”aerosol injection into the atmosphere” or similar wording, we only seach for ”aerosol injection”.
SRM	aerosol injection	(stratospheric OR atmosphere OR stratosphere) (aerosol OR sulfate OR sulphate) (injection OR inject OR spraying OR scattering)	5963	Query to capture tweets that discuss SAI activities without mentioning the technique explicitly.
SRM	cloud brightening	”cloud brightening”	4117	Cloud brightening refers to techniques that increase the albedo or seed clouds, mostly in marine environments.
SRM	cloud brightening	”cloud albedo enhancement”	30	Synonym for ”cloud brightening”
SRM	cloud brightening	”marine sky brightening”	10	Synonym for ”marine cloud brightening”

Continued on next page

category	technology	subquery	count	explanation
SRM	cloud brightening	"marine cloud" (seeding OR engineering)	79	Often, "cloud brightening" techniques are discussed in the context of marine cloud cover.
SRM	surface albedo modification	"albedo modification"	726	Albedo modification refers to techniques to increase the surface reflectivity of the earth, e.g. by whitening surfaces in the built environment.
SRM	surface albedo modification	albedo (earth OR climate OR surface) (modify OR increase)	876	As many tweets on "albedo" refer to a game of the same name, we specified this query to capture mentions of increasing albedo.
SRM	surface albedo modification	"surface-based brightening"	5	Synonym of "albedo modification"
SRM	surface albedo modification	"high albedo" (crop OR building)	20	Albedo modification is discussed especially for buidlings and agricultural landscapes by modifying crops.
SRM	cloud thinning	"cirrus thinning"	24	"Cirrus (cloud) thinning" refers to techniques to reduce cirrus cloud cover which absorbs infrared radiation from the earth.
SRM	cloud thinning	"cirrus cloud" (thinning OR seeding)	214	Query to capture other mentions of cirrus cloud thinning.
SRM	space shades	"space sunshade" OR "sunshade in space"	189	Space sunshades are proposed structures in space to shield parts of the sunlight. As tweets on "space mirror" did not discuss SRM techniques but mirrors of actual space telescopes.

Continued on next page

category	technology	subquery	count	explanation
SRM	space shades	space shade (earth OR planet) (cloth OR tarpet OR sheet OR "climate change" OR warming)	130	Query to cover mentions of space shading techniques, modified to not capture climate adaptation measures.
SRM	space shades	"space parasol" OR "parasol in space"	246	Synonym for "space sunshade"
SRM	space shades	(orbit OR orbital) (sunshield OR shade OR sunshade OR reflector) (planet OR climate OR global)	160	
SRM	space shades	("space sunshield" OR "sunshield in space") -webb	30	Synonym for "space sunshade", excluding mentions of the sunshield of the Webb telescope.
GGR	GGR (general)	"negative emissions"	36943	Greenhouse gas removal techniques are often also referred to as "negative emission(s)".
GGR	GGR (general)	"negative emission"	3715	Greenhouse gas removal techniques are often also referred to as "negative emission(s)".
GGR	GGR (general)	"carbon dioxide removal"	10039	Greenhouse gas removal for CO2
GGR	GGR (general)	"co2 removal" -submarine -"space station"	11102	Synonym for "carbon dioxide removal", excluding tweets that refer to air treatment in submarine or space vessels.
GGR	GGR (general)	"carbon removal"	34126	Synonym for "carbon dioxide removal".

Continued on next page

category	technology	subquery	count	explanation
GGR	GGR (general)	"greenhouse gas removal"	2146	"Greenhouse gas removal" refer to all kinds of activities or techniques that can remove anthropogenic greenhouse gases from the atmosphere.
GGR	GGR (general)	"ghg removal"	401	Acronym for "greenhouse gas removal"
GGR	GGR (general)	"carbon negative" (climate OR co2 OR emission OR "greenhouse gas" OR ghg)	11577	"Carbon negative" often paraphrases techniques to remove CO2 from the atmosphere.
GGR	GGR (general)	(remove OR removing OR removed) (carbon OR co2) atmosphere	35439	Query to also capture CO2 removal without naming the specific terms above.
GGR	methane removal	"methane direct air capture"	2	Methane removal usually refers to techniques that directly remove methane from the atmosphere.
GGR	methane removal	"methane capture"	3338	
GGR	methane removal	methane removing atmosphere	208	
GGR	methane removal	"methane removal"	512	
GGR	ocean fertilization	"ocean fertilization" OR "ocean fertilisation"	3244	Ocean fertilization is a technique to induce algal blooms that capture CO2.
GGR	ocean fertilization	"iron fertilization" OR "iron fertilisation"	5133	Iron is often the limiting nutrient, which is why iron is commonly used to fertilize parts of the ocean.

Continued on next page

category	technology	subquery	count	explanation
GGR	ocean fertilization	(fertilization OR fertilisation) (phytoplankton OR algae) (climate OR carbon OR co2)	232	Query to capture other mentions of iron fertilization without using the term directly.
GGR	ocean fertilization	"iron seeding" (climate OR co2 OR carbon)	162	Iron seeding is sometimes used as a synonym to iron fertilization. As it can also refer to other things, tweets also need to mention climate related keywords.
GGR	ocean alkalinization	"ocean liming" -from:spangletoes	88	Synonym of ocean alkalinity enhancement. A large percentage of tweets with this keyword were spam from one user and were excluded.
GGR	ocean alkalinization	"ocean alkalinity enhancement"	182	Ocean alkalinity enhancement refers to processes that bind ocean CO2 in carbonates.
GGR	enhanced weathering	"enhanced weathering" -from:spangletoes	1735	Enhanced weathering refers to techniques that bind CO2 chemically using ground rock. A large percentage of tweets with this keyword were spam from one user and were excluded.
GGR	enhanced weathering	(olivine OR basalt OR silicate) (co2 OR emission OR emissions)	4653	Rocks used for enhanced weathering are usually olivine, basalt or silicate rocks.
GGR	enhanced weathering	olivine weathering	659	

Continued on next page

category	technology	subquery	count	explanation
GGR	enhanced weathering	(basalt OR silicate) weathering (co2 OR carbon OR enhanced)	550	As many tweets on basalt or silicate weathering refer to natural processes, they also need to name enhancement or CO2 explicitly.
GGR	biochar	(biochar OR bio-char) (co2 OR carbon OR climate OR emission OR sequestration OR "greenhouse gas")	19312	Biochar can be used to store carbon of atmospheric origin for example in soils with many co-benefits. This query only considered tweets that related biochar in some way to climate or CO2.
GGR	afforestation and reforestation	afforestation (climate OR co2 OR emission OR emissions OR "greenhouse gas" OR ghg OR carbon)	9440	Afforestation is the practice of planting a new forest in an area that was previously not forrested. This query only considers tweets that also mention climate-related keywords.
GGR	afforestation and reforestation	reforestation (climate OR co2 OR emission OR emissions OR "greenhouse gas" OR ghg OR carbon)	38484	Reforestation is the practice of replanting a trees in a previously deforested area. This query only considers tweets that also mention climate-related keywords.
GGR	afforestation and reforestation	tree planting climate	35741	Tree planting for climate
GGR	ecosystem restoration	(re-wilding OR rewilding) (climate OR carbon OR CO2 OR "greenhouse gas" OR GHG)	19808	Rewilding of

Continued on next page

category	technology	subquery	count	explanation
GGR	Soil carbon sequestration	soil sequestration (co2 OR carbon)	15166	Soil carbon sequestration refers to techniques that enhance the organic carbon in soils.
GGR	Soil carbon sequestration	"soil carbon"	51058	
GGR	Soil carbon sequestration	"carbon farming"	32849	Carbon farming is used to describe techniques to enhance the carbon content in soils.
GGR	BECCS	BECCS (co2 OR carbon OR climate OR ccs OR biomass OR emission OR emissions)	11393	BECCS is an acronym for "bioenergy with carbon capture and storage". As it is also used as a nickname, the query also requires mention of climate or energy related keywords.
GGR	BECCS	biomass ("carbon capture" OR "capture carbon" OR "co2 capture" OR "capture CO2" OR ccs)	6069	Combination of biomass with different combinations describing the capture and storage component.
GGR	BECCS	bioenergy ("carbon capture" OR "capture carbon" OR "co2 capture" OR "capture CO2" OR ccs)	5821	Combination of bioenergy with different combinations describing the capture and storage component.
GGR	blue carbon	seagrass (carbon OR co2)	11049	Blue carbon includes the capture of CO2 by seagrass.
GGR	blue carbon	macroalgae (carbon OR co2)	460	Blue carbon includes the capture of CO2 by macroalgae.
GGR	blue carbon	mangrove (carbon OR co2)	10260	Blue carbon includes the capture of CO2 by mangroves.
GGR	blue carbon	kelp (carbon OR co2)	7015	Kelp is a synonym for macroalgae.

Continued on next page

category	technology	subquery	count	explanation
GGR	blue carbon	(wetland OR wetlands OR marsh OR marshes OR peatland OR peatlands OR peat OR bog OR bogs) (carbon OR co2) (restore OR restoration OR rehabilitation)	5513	Blue carbon also includes carbon sequestration by wetland, marsh or peatland restoration.
GGR	blue carbon	"blue carbon" -"carbon fiber" - "carbon fibre" -"carbon roof" -ring -ferrari -lamborghini -dodge -bugatti - adidas -nike	29393	Blue carbon refers to carbon removal and sequestration in marine ecosystems. As this search term also returns some product description with blue carbon fibers, we excluded these from the query.
GGR	direct air capture	DAC (climate OR carbon OR co2 OR emission OR emissions)	7749	DAC is the acronym for "direct air capture". As it can also stand for other things, the tweet needs to also mention climate-related keywords.
GGR	direct air capture	"direct air capture"	14822	Direct air capture refers to artificial chemical processes to capture atmospheric CO2. This is complemented by a storage component to permanently remove CO2 from the atmosphere.

Continued on next page

category	technology	subquery	count	explanation
GGR	direct air capture	(”carbon capture” OR ”co2 capture”) (”ambient air” OR ”direct air”)	3073	This query gets tweets that refer to DAC(CS) but do not use the term directly.
GGR	direct air capture	DACCS (carbon OR co2 OR climate)	428	DACCS stands for ”direct air carbon capture and storage”.
CCS	CCS	”co2 sequestration” storage	212	
CCS	CCS	”carbon sequestration” storage	1886	
CCS	CCS	”carbon dioxide sequestration”	803	
CCS	CCS	”carbon capture” storage	102607	”Carbon capture and storage” usually refers to techniques to capture CO2 from exhaust gases from fossil fuel plants and store it underground. As there are variants, we search for different combinations of the terms.
CCS	CCS	”carbon storage” capture	1790	
CCS	CCS	”carbon dioxide capture” storage	1096	
CCS	CCS	”carbon dioxide storage” capture	998	
CCS	CCS	CCS (climate OR carbon OR co2)	97521	As CCS is also an acronym for other things, the query needed to be specified to also include climate related terms.

S3 Filter for conspiracy-related tweets

We mark a tweet as conspiracy-related if either one of the strong keywords is matched in the text or two of the weak keywords.

Strong keywords: wedonotconsent, chemtrail, chemtrails, haarp, ChemPlanes, elites, skybasterds, deathfromabove, OpChemtrails, RealGeoEngWatch, LookUp, Qanon, QArmy, sheep, Wigington, 5GTakeover, (Geoengineering AND Watch AND Global AND Alert), DeepState, WeatherManipulation, infowars.com, killingUsAll, StoptheMadnessNow, climateviewer, Warfare, wakeup, agenda21, BigPharmaLies, Bilderberg, GeoChemFall
Weak keywords: poison*, toxic, aluminum, gates, kill*, depopulat*, (sky OR skies), spray*, expos*, scam, lies, chemical*, covert*

If a tweet has been classified as conspiracy-related this does not mean that the tweet is supporting a conspiracy theory, it could also be that it is mentioning or refuting such a theory. However, from manual inspection, most of the conspiracy-related tweets either support or mention conspiracies without refutation. We designed the filter to give a lower estimate of conspiracy related tweets. It has a high precision on our set of 300 manually annotated tweets (94%) but a low recall (62%).

References

Caldeira, K., G. Bala, and L. Cao (May 2013). "The Science of Geoengineering". en. In: *Annual Review of Earth and Planetary Sciences* 41.1, pp. 231–256. DOI: 10.1146/annurev-earth-042711-105548.

Cummings, C. L., S. H. Lin, and B. D. Trump (Sept. 2017). "Public perceptions of climate geoengineering: a systematic review of the literature". en. In: *Climate Research* 73.3, pp. 247–264. DOI: 10.3354/cr01475.

Minx, J. C., W. F. Lamb, M. W. Callaghan, S. Fuss, J. Hilaire, F. Creutzig, T. Amann, T. Beringer, W. de Oliveira Garcia, J. Hartmann, T. Khanna, D. Lenzi, G. Luderer, G. F. Nemet, J. Rogelj, P. Smith, J. L. Vicente Vicente, J. Wilcox, and M. del Mar Zamora Dominguez (May 2018). "Negative Emissions—Part 1: Research Landscape and Synthesis". In: *Environmental Research Letters* 13.6, p. 063001. DOI: 10.1088/1748-9326/aabf9b.

Vaughan, N. E. and T. M. Lenton (Dec. 2011). "A review of climate geoengineering proposals". en. In: *Climatic Change* 109.3, pp. 745–790. DOI: 10.1007/s10584-011-0027-7.
